# Toward Drug-Like Multispecific Antibodies by Design

**DOI:** 10.3390/ijms21207496

**Published:** 2020-10-12

**Authors:** Manali S. Sawant, Craig N. Streu, Lina Wu, Peter M. Tessier

**Affiliations:** 1 Department of Pharmaceutical Sciences, University of Michigan, Ann Arbor, MI 48109, USA; manalis@med.umich.edu (M.S.S.); cstreu@albion.edu (C.N.S.); 2 Biointerfaces Institute, University of Michigan, Ann Arbor, MI 48109, USA; linawu@umich.edu; 3 Department of Chemistry, Albion College, Albion, MI 49224, USA; 4 Department of Chemical Engineering, University of Michigan, Ann Arbor, MI 48109, USA; 5 Department of Biomedical Engineering, University of Michigan, Ann Arbor, MI 48109, USA

**Keywords:** bispecific, polyspecificity, pharmacokinetics, solubility, aggregation, viscosity, developability, stability, affinity, specificity, protein engineering, self-association, non-specific binding, immunogenicity

## Abstract

The success of antibody therapeutics is strongly influenced by their multifunctional nature that couples antigen recognition mediated by their variable regions with effector functions and half-life extension mediated by a subset of their constant regions. Nevertheless, the monospecific IgG format is not optimal for many therapeutic applications, and this has led to the design of a vast number of unique multispecific antibody formats that enable targeting of multiple antigens or multiple epitopes on the same antigen. Despite the diversity of these formats, a common challenge in generating multispecific antibodies is that they display suboptimal physical and chemical properties relative to conventional IgGs and are more difficult to develop into therapeutics. Here we review advances in the design and engineering of multispecific antibodies with drug-like properties, including favorable stability, solubility, viscosity, specificity and pharmacokinetic properties. We also highlight emerging experimental and computational methods for improving the next generation of multispecific antibodies, as well as their constituent antibody fragments, with natural IgG-like properties. Finally, we identify several outstanding challenges that need to be addressed to increase the success of multispecific antibodies in the clinic.

## 1. Introduction

Antibodies are among the most well-established biologics and are widely employed as therapeutics. Their success as therapeutics is largely due to their unique combination of properties, including their favorable activities, safety profiles, and physical and chemical properties (also known as developability properties). The activity of antibodies is linked to their high binding affinities and specificities as well as their Fc-mediated interactions with receptors that enable extended half-lives and, in some cases, effector functions. The safety of antibodies is due to their low off-target binding, low immunogenicity (for human or humanized antibodies), and non-toxic breakdown products (amino acids). The desirable developability properties of antibodies are due to their high folding stabilities, high solubilities, low viscosities and high chemical stabilities. The combination of these key properties has led to >80 approved antibody drugs and hundreds more in clinical trials [1].

**Figure 1:**
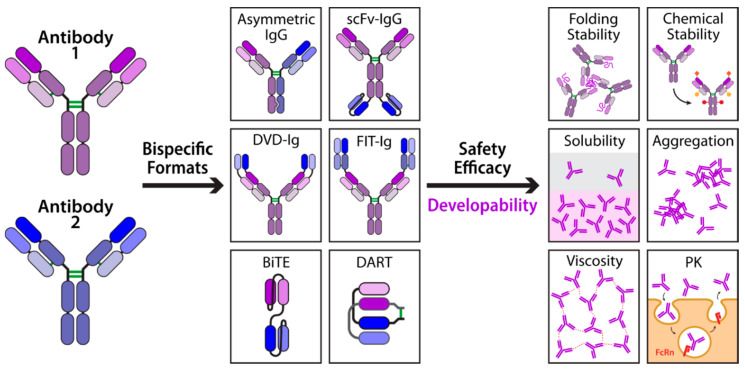
Antigen-binding regions of monospecific IgGs can be combined into various multispecific formats that have unique developability concerns, including those related to their stability, solubility, aggregation, viscosity, and pharmacokinetics. The abbreviations are scFv-IgG for single-chain variable fragment immunoglobulin, DVD-Ig for dual variable domain immunoglobulin, FIT-Ig for fabs-in-tandem immunoglobulin, BiTE for bispecific T-cell engager and DART for dual-affinity re-targeting antibody fragment.

Nevertheless, most lead antibody (IgG) candidates do not have the required combination of activity, safety, and developability properties for therapeutic applications, and must be further engineered to achieve drug-like molecules. Overcoming these issues is even more challenging for multispecific antibodies, a class of engineered antibodies that seeks to engage either two or more targets or two or more epitopes on the same target. Generally, multispecifics are chimeric proteins composed of IgGs and smaller antibody fragments or multiple antibody fragments (Figure 1). Given their ability to bind more than one target, multispecific antibodies have functional advantages for applications in which it is necessary to bring together two targets in close proximity. This is a key feature of the two multispecific antibodies blinatumomab and emicizumab that have been approved for use in humans and are currently being marketed [2,3,4,5,6]. Although the full potential of multispecific antibodies is just beginning to be tapped [7], one particularly notable therapeutic application has been the recruitment of immune effector cells, such as T cells [8,9,10,11] or natural killer (NK) cells [12,13,14,15], to tumor cells using multispecific formats that recognize antigens on both cell types. Still other potential advantages of binding multiple targets include synergistic effects, improved specificity, and reduced incidence of drug-resistance.

Despite their impressive therapeutic potential, much less is known about the developability properties of multispecifics with respect to conventional antibodies (IgGs), which is a major limitation to their development and broad adoption as therapeutics. Further exacerbating the knowledge gap is the sheer number of multispecific antibody formats that have been developed, each with its own specific advantages and liabilities as therapeutics [16,17,18]. Recent advances in the ability to screen antibodies for developability parameters earlier in the discovery process are likely to streamline the development of antibody therapeutics but, to date, no such general approach exists for multispecific antibodies. Because many multispecific antibodies are composed of recombined antibody fragments, and stabilization of the individual fragments is a common component of multispecific design strategies, this review will focus on the latest methods for optimizing the drug-like properties of antibody fragments in addition to strategies for optimizing multispecific antibodies. Here we divide the critical antibody properties into three broad categories, namely: (i) physical and chemical stability, (ii) self-association and high concentration properties, and (iii) polyspecificity and in vivo properties. For each category, we highlight the latest advances in understanding how to design and engineer multispecific antibodies and their constituent antibody fragments with drug-like properties as well as outline the outstanding challenges.

## 2. Physical and Chemical Stability

### 2.1. Folding and Assembly

Many new multispecific formats are generated from constituent antibody fragments. Typically, the folding stability of these fragments is lower than that for full-length IgGs, which can be problematic following incorporation into multispecific formats. Much previous work has focused on the thermostabilization of antibody fragments and full-length antibodies [19,20,21,22], while comparatively less is known about the thermostability of diverse multispecific formats [23]. However, it is possible to improve the stability of multispecific antibodies by optimization of their component parts. Therefore, thermostabilization of antibody fragments is an important strategy for producing multispecific antibodies with improved overall stability.

One established method for improving the thermostability of antibody fragments is disulfide engineering. The addition of well-positioned disulfide bonds is known to improve the thermostability of antibodies and fragments thereof, and the number of engineered disulfide bond variants validated for stabilization, as well as the list of successful applications, continues to expand [24,25,26,27]. Disulfide engineering has been particularly successful in generating stabilized single-domain antibodies with one recent example generating an extremely stable nanobody with a melting temperature exceeding 90 °C [28,29,30,31]. The ability to generate such highly stable single-domain antibodies is likely to shift the developmental bottleneck for multispecifics that contain these fragments away from thermal stabilization of the constituent fragments toward optimization of other developability properties.

One such property is proper folding. Despite the success of disulfide engineering in protein stabilization, introducing additional non-canonical disulfide bonds to proteins, such as scFvs, comes at the risk of scrambled or mismatched disulfides. This scrambling can lead to incorrect folding or multimerization, resulting in lost production yield and purity [32]. Therefore, when considering disulfide engineering as an approach to antibody fragment thermostabilization, the potential stabilizing effects must be weighed against the potential for disulfide scrambling and misfolding. In multispecific formats that may contain increased numbers of disulfide bonds, the potential for mispairing may be magnified. Fortunately, novel approaches for improving the yield of proteins with engineered disulfide bonds have been investigated [33]. One particularly powerful method is the co-expression of four proteins that promote folding and disulfide bond formation in the periplasm of *E. coli* from a single helper plasmid [34]. These helper proteins include DsbA and DsbC, which are thiol-disulfide oxidoreductases that are responsible for the reduction and subsequent rearrangement of improperly formed disulfide bonds. In addition, the other two proteins include peptidyl-prolyl *cis*/*trans*-isomerases FkpA and SurA. These enzymes promote sampling both *cis* and *trans* isomeric forms of proline during protein refolding. Application of this technology to nanobodies containing a second stabilizing disulfide bond has been reported to increase expression yield by 2- to 10-fold, approaching and occasionally exceeding the expression yields for nanobodies containing a single canonical disulfide bond [35].

Given the added risk of disulfide scrambling and subsequently reduced yields associated with disulfide engineering, other approaches for antibody fragment thermostabilization are of broad interest. One strategy for generating antibody fragments with high stability is to graft portions of a known antibody or antibody fragment, especially the CDRs, onto a thermostable scaffold [22,36]. However, the affinity and stability of an antibody or antibody fragment is generally highly dependent upon the compatibility of the framework and CDR regions since CDRs make many contacts that can drastically alter these properties [37,38]. This means that grafted CDR regions or affinity-improving mutations cannot be incorporated into a thermostable framework without risk of compromising antibody stability. Although compensatory mutations can offset this loss of stability, iterative rounds of affinity and stability maturation are generally required [21,22,37,39]. A related strategy that involves thermostabilization of the framework has recently been demonstrated that involves V_L_ framework swapping [40]. The investigators identified a suboptimal lambda V_L_ domain in an anti-epidermal growth factor receptor (EGFR) scFv and replaced it with more stable kappa3 framework regions, generating a scFv variant with greater stability, improved expression in *E. coli*, and similar binding to the parental antibody fragment.

Yet another method for generating thermally stable antibodies is the use of high-throughput biophysical profiling. Among the most common strategies for selecting stabilized antibody fragments is the use of thermal challenge assays of libraries displayed on phage or yeast [41,42,43,44,45]. In these assays, libraries are heat shocked before selection for antigen binding. This strategy was recently used successfully to improve the stability of a bispecific tetravalent antibody by allowing the rapid optimization of its scFv subunits [46]. Of course, this type of assay relies on the viability of the display system following heat shock, which has led to the development of a new thermostable yeast strain for use in such heat shock screens [47]. Although this fragment-based approach has proven effective, recent advances in screening intact bispecific antibodies suggests that the final format of an antibody can have unexpected consequences for potency, and may therefore identify variants with optimal properties that may be missed by the fragment-based approaches [48].

Despite the success of these and other approaches [47,49,50,51,52], stabilizing mutations in the CDRs or frameworks would be highly valuable. Fortunately, computational approaches for the design of antibodies with high affinity and thermostability have made significant recent advances [53,54,55,56,57,58]. One notable method also approaches the problem by computationally recombining antibody variable domains to generate variants with improved biophysical properties [53]. Specifically, stabilized antibodies against multiple antigens were designed by a computational algorithm known as AbDesign [53,59]. The AbDesign algorithm optimizes both binding and stability by first generating recombinations of known F_V_ backbone fragments, which are docked against a desired target. Next, affinity and stability are simultaneously optimized by sampling different natural conformations of the F_V_ backbone fragments and optimizing their sequences using Rosetta. The corresponding scFvs could then be further affinity maturated into nanomolar binders experimentally using error-prone PCR and in vitro library sorting methods. After five rounds of design, the resulting antibody fragments contained more than 30 backbone mutations with respect to the mammalian germline sequence and achieved respectable folding stabilities, as measured in terms of their melting temperatures (57–79 °C relative to 70 °C for similar wild-type constructs). To achieve this level of affinity and stability, the authors conclude that it is critical to both maintain residues required for proper F_V_ backbone configurations, such as buried polar networks, and segment the F_V_ backbone fragments during the design process in a manner that keeps the CDRs and their closest framework contacts on the same fragment. Using those constraints, the authors suggest that their fragmentation strategy is a generally applicable approach to computational design of stabilized antibodies, which will be important to further evaluate in future studies.

**Figure 2:**
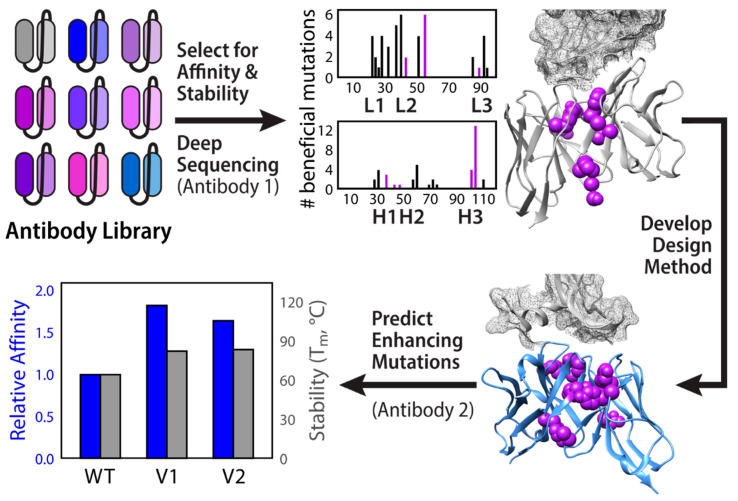
Evaluation and prediction of mutations in the V_H_/V_L_ interface that enhance the stability and affinity of single-chain antibodies. Deep mutational analysis of a scFv library (top left) identified eight interface residues tolerant of mutations (purple, top right). Combinations of affinity-maintaining mutations at these sites were analyzed with Rosetta to predict those that also improve thermal stability. After development using the anti-lysozyme antibody D44.1, the automated design method (AbLift) was validated on two additional antibodies, including the anti-VEGF antibody G6 (bottom right). AbLift designed G6 variants 1 (V1) and 2 (V2) displayed both improved affinity and thermal stability (bottom left). The figure is adapted from a previous publication [60].

Another streamlined computational approach to antibody fragment affinity maturation and stabilization focuses on optimization of the V_H_-V_L_ interface (Figure 2) [60]. This strategy was inspired by deep mutational analysis data for anti-lysozyme antibody D44.1 suggesting that, in addition to the CDRs, many affinity-enhancing mutations occur at the V_H_-V_L_ interface. From this mutational data, Rosetta was used to generate optimized multipoint mutants from combinations of the affinity-enhancing mutations at eight interface positions. This method yielded an antibody with nearly ten-fold greater affinity than wild type with substantially improved thermal stability (melting temperature increase of 9 °C relative to wild type) and aggregation resistance (aggregation temperature increase of 7 °C relative to wild type). Comparison of the X-ray structures of the wild type and designed Fabs showed a high degree of structural similarity in the lysozyme binding site. Furthermore, the designed antibody had a 25-fold slower off rate than the parental antibody. Taken together, these results suggest that the improvements in affinity are likely a result of improved stabilization of the binding conformation, as well as improved packing, solvation and/or rigidity in the resulting framework backbone. Therefore, optimization of the V_H_-V_L_ interface has the potential to improve binding and developability properties in a number of ways. This method was then automated into a freely available web-based platform known as AbLIFT, which was validated on two additional antibodies, both of which showed substantial improvements in affinity, thermal stability, and aggregation resistance [60]. Recently, a related strategy was employed for scFv and single-chain antibody (scAb) formats, placing special emphasis not necessarily on domain interfaces, but on local regions that are particularly susceptible to unfolding [57]. Once again, the application of Rosetta to determine multipoint mutants with enhanced properties within a defined region yielded significant enhancements in thermal stability, suggesting that these approaches are not necessarily format specific and therefore hold potential for engineering multispecific antibodies.

Although multispecific antibodies face their own challenges associated with thermal stability, their proper and efficient assembly into relatively large and complex molecular formats is one of the greatest barriers to their successful development into viable therapeutics. In traditional antibody-like formats, this includes proper heavy chain pairing as well as heavy-light chain pairing. These assembly issues have generally impaired high-throughput study of multispecific properties, slowing their development.

Multiple approaches for the efficient production of multispecific antibodies have recently been reported, including those that range from improved purification procedures [61] to novel strategies for reducing mispairing [62,63,64,65,66]. These later strategies can broadly be divided into two categories: novel multispecific formats that are less prone to mispairing and new strategies for assembling multispecifics. For example, common light chain approaches have been developed to address the light chain pairing problem while generating different F_v_ antigen-binding sites using divergent heavy chains [67,68,69]. Given that the light chains for each binding site are the same, this eliminates the possibility of heavy chain-light chain mispairing. However, identifying a promiscuous light chain that enables binding of two different antigens can be a significant undertaking. Still others have engineered the heavy chain-light chain interface to promote selective association of the correct heavy chain-light chain pairs, or perhaps just as importantly, disfavor incorrect chain pairing [70,71,72,73]. This type of engineering has the potential to allow direct expression and production of heterodimeric antibodies, which are antibodies composed of half of two different antibodies dimerized into a full IgG through heterotypic Fc pairing [74,75]. Given that the engineered sites are buried at antibody domain interfaces, these frameworks are anticipated to exhibit minimal immunogenicity, which is an important consideration for their viability as potential therapeutics [67].

Assembly issues notwithstanding, multispecific antibody formats have the potential to show highly favorable drug-like properties. For example, the Bispecific T-Cell Engager (BiTE) format, which is composed of two scFvs linked in sequence, was the first bispecific drug format to earn FDA approval [76]. By making use of the propensity of scFvs to atypically heterodimerize through intermolecular disulfide formation, another related bispecific antibody fragment (Dual-Affinity Re-Targeting, DART) has been produced [77]. These bispecific antibody fragments, which are composed of sets of heterodimerized scFvs that are stabilized by interstrand disulfide bonds, have shown even greater bioactivity and stability than the equivalent BiTE format [78,79]. Other recent additions to the growing list of multispecific formats include bivalent tandem Fabs [80], as well as the related tetravalent bispecific IgG1-Fabs [80] and Fabs-in-tandem (FIT-Ig) [81]. Unlike BiTEs that are single-chain antibodies, or DARTs that are composed of two chains, all three of these formats are expressed as three chains. IgG1-Fabs contain a Fab recombinantly fused to a parent antibody V_H_ through the Fab C_H_1 with a linker. On the other hand, Fabs-in-tandem are composed of two Fabs linked from the V_H_ of one Fab to the C_L_ of another (instead of to C_H_1 for IgG1-Fabs), leading to a “crisscross” Fab orientation. Bivalent Fabs-in-tandem composed of anti-EGFR and anti-CD3 Fabs showed excellent antigen binding and superior thermostability, aggregation propensity and expression compared to the corresponding tandem scFv, demonstrating its potential as a multispecific scaffold [81]. Similar improvements in stability, aggregation, and expression were observed for the IgG1-Fab constructed from the Fab of pertuzumab fused to the IgG1 of trastuzumab relative to the corresponding IgG1-scFv [80]. It is worth noting that both formats demonstrated excellent bioactivity and that the IgG1-Fab also showed improved bioactivity with respect to the combination of parental antibodies, further highlighting the unique therapeutic potential of multispecific formats. Such bispecific antibodies composed of the anti-IL-17 Fab of ixekizumab fused to the anti-IL-20 antibody 15D2 showed little mispairing during assembly except for a small quantity of light chain dimerization [81]. Like the other recent Fab-based multispecific formats [80,82], these Fabs-in-tandem displayed excellent biophysical properties [81].

While these new antibody formats have significant advantages, each new antibody format brings with it uncertainties about drug-like properties and developability. Therefore, multispecific antibodies with native IgG frameworks are still considered most desirable. Recently, intein-based protein splicing methods have proven highly effective for generating multispecific antibodies from antibody fragments [83,84] as well as native IgG frameworks [85,86]. Although the full impact of this technology has yet to be realized, the ability to make multispecifics with fully native IgG Fc regions will undoubtedly have significant implications for multispecific antibody production for a variety of reasons. For example, this intein approach will enable generation of complex multispecific antibodies in a much higher throughput fashion than is currently possible. Such methods will be necessary for generating early-stage developability data to assess the biophysical properties of multispecifics, which is one area where multispecifics lag far behind traditional monospecific antibody formats. Fortunately, the unique advantages of intein splicing have already been applied in a generalizable high-throughput screen for bispecific antibodies, demonstrating the applicability of the technology for screening applications [87].

### 2.2. Aggregation

Aggregation is a particularly important liability for biotherapeutic drug development because it can impact all stages of production and, therefore, strategies for reducing aggregation are an important part of antibody design efforts. For example, high levels of aggregate formation during protein expression and cell culture can cause low purification yields of the antibody of interest [32]. Even more important, aggregates can also form during formulation and storage, and dimers, trimers, and higher order multimers can lead to undesirable immunogenic responses [88,89].

Although aggregation is commonly associated with reduced folding stability, leading to unfolding or misfolding, these properties are not always linked. For example, we and others have shown that antibody fragments can display large differences in their aggregation propensities despite having similar folding stabilities [90,91,92,93]. Additionally, it is difficult to identify a single attribute that can be used to predict protein aggregation due to the complexities of the locations of aggregation-prone regions in antibodies and other proteins, as some are solvent-exposed in the folded state and others become solvent exposed upon unfolding [94,95]. Moreover, the propensity of antibody fragments to aggregate may be amplified by avidity effects when reformatted as multispecifics or in conditions that may be encountered during bioprocessing, such as freeze-thaw cycles, pH changes or mechanical stresses such as shaking and stirring [94,96,97].

Several powerful methods have been developed recently to experimentally and computationally identify antibody variants that are resistant to aggregation [98,99]. One general strategy is to modify the charge of antibodies or antibody fragments. Applications that address the aggregation of antibody fragments are important because a significant challenge in developing multispecific antibodies as drugs is that the constituent antibody fragments used in common multispecific antibody formats are generally more prone to aggregation than conventional IgGs. The most common approach to suppress antibody aggregation is to replace hydrophobic residues with charged residues, which generates aggregation-resistant variants by reducing hydrophobic association between species [92,100]. For example, phage display has been used for mutational screening of CDR1 and CDR2 in human variable domains to determine the contribution of individual residues to aggregation [92]. This analysis revealed a set of seven positions localized in or near CDR1 of V_H_ and six positions localized in or near CDR2 of V_L_ that were responsible for the greatest contributions to the aggregation of human antibody variable domains. Mutational analysis was also used to show that inclusion of aspartate or, less favorably, glutamate residues at these positions resulted in substantially reduced aggregation behavior. Interestingly, the results suggested that the contribution of each position were generally independent and qualitatively additive, such that multiple mutations led to the highest levels of aggregation resistance. Not only did these substitutions improve aggregation behavior, they also resulted in generally improved biophysical properties. Surprisingly, an earlier study of the same aggregation-prone V_H_ domain identified a novel negatively-charged mutation near the edge of heavy chain CDR1 (F29D) that was as effective at preventing aggregation as multiple negatively-charged mutations in the middle of the same CDR [90]. It was also demonstrated that residues near the edges of CDR3 in human single-domain antibodies contribute disproportionately to aggregation and that the most effective mutations for reducing aggregation vary with respect to the overall charge of the antibody fragment [93]. A similar mutational strategy was used to improve the solubility and aggregation profile of a F(ab’)_2_ [100]. Interestingly, the sites that were found to be critical for these improvements were located along the perimeter of the paratope. This finding, which is similar to those for other types of protein-protein interactions [101,102,103], may explain the results of the mutational analysis and influence future design strategies.

While these mutational strategies have proven highly effective at improving the aggregation behavior of antibodies and antibody fragments, it would ideally be possible to select for aggregation-resistant variants earlier in the development process and with higher accuracy. Important progress has been made in recent years to accomplish this ambitious goal by combining these mutational strategies with computational predictive methods. For example, using previously identified sites in antibody variable regions that mediate aggregation [92], an in silico library of 393 single, double, and triple aspartate mutants were generated and screened using Rosetta to identify those that retain thermodynamic stability [104]. Those mutants predicted to retain high thermodynamic stability were produced and tested for various developability attributes. Of the 26 variants produced, half showed reduced aggregation, while nearly all (25 of 26) demonstrated reduced non-specific binding. Interestingly, the judiciously identified mutations had a negligible impact on antigen-binding affinity, demonstrating the tremendous potential of this approach for streamlining antibody development.

One potential consequence of mutational design strategies that alter antibody charge is that added surface charge may increase transient local unfolding, which could lead to an increase in aggregate nucleation [105]. To investigate this, the electrostatic potential of a model scFv was systematically modified by insertion of three different sets of five charged residues (Glu, Lys or Arg residues) into a hydrophobic patch in such a way as to introduce new salt bridges [106]. In all cases, replacing arginine with lysine was shown to reduce aggregation, leading to the conclusion that lysine has an increased ability, relative to arginine, to reduce interactions between partially folded states, which may become more prominent after electrostatic engineering. More recently, the biophysical basis of this finding was shown to be a result of the propensity of arginine to form favorable interactions with the unfolded states of proteins, possibly through interactions with exposed aromatic residues [107]. Furthermore, the unique geometric structure afforded by the guanidinium group in arginine permits multiple types of intermolecular interactions and thus can promote aggregation [108]. As such, the authors conclude that the arginine:lysine ratio may be a useful parameter for predicting antibody aggregation propensities and for designing antibody variants with reduced aggregation behavior [107].

Several additional notable studies in predicting aggregation behavior have focused on single-chain (scFv) antibodies, which are one of the most aggregation-prone antibody fragments. This is due in large part to the relatively weak interactions between V_H_ and V_L_ domains in the absence of the stabilizing constant (C_H_1 and C_L_) domains, which results in a dynamic equilibrium between open and closed forms of the fragment. This process facilitates the formation of intermolecular domain swapping and high levels of aggregation. Rational design and high-throughput mutagenesis methods have both been used successfully to reduce aggregation by improving the stability of the V_H_/V_L_ interface and promoting the closed state of the scFv [40,109,110,111,112,113].

Although interface stabilization strategies have proven effective, a broadly generalizable approach for aggregation reduction across many different types of scFv frameworks would be highly desirable. Interestingly, intramolecular chain cyclization of scFvs has been shown to be effective at reducing the propensity of scFvs to dissociate and subsequently aggregate [114]. Specifically, sortase-cyclized scFvs demonstrated an increased fraction of closed states as well as reduced separation between V_H_ and V_L_ domains in the open states. In addition, these improvements came at little cost to the overall thermostability and antigen binding. This strategy was also demonstrated to be generalizable to other scFvs. However, cyclic scFv fragments cannot be incorporated into traditional multispecific frameworks such as IgG-scFv formats in the same ways as the conventional (acycylic) versions, necessitating the development of novel strategies for linking cyclic scFvs into multivalent therapeutics.

As advances in assays for aggregation have improved, so too have high-throughput methods for analyzing aggregation propensity early in development [92,115]. Two particularly notable studies have sought correlations between early-stage analyses of biophysical parameters and late-stage developability [116,117]. In the first study, the results of twelve different biophysical assays for 137 antibodies that had advanced to at least phase II clinical studies were analyzed [116]. This information was used to correlate biophysical assay results, many of which can be performed in a high-throughput fashion early in the discovery process, to drug-like properties and to establish thresholds for each of these properties. Interestingly, the results from multiple assays could be correlated with each other, such as strong correlations between different types of non-specific and self-interaction assays. However, accelerated stability (aggregation) results could not be quantitatively correlated with the results from other assays, highlighting the distinctive nature of antibody aggregation and the difficulty in assessing aggregation behavior using early-stage screens [116].

In the second related study, the results of a largely different set of early-stage biophysical assays for 152 human or humanized monoclonal antibodies were evaluated for correlations between early-stage screening results and downstream developmental parameters [117]. The authors observed a significant number of correlations between early-stage high-throughput assays and later stage analytical methods that often require much more sample. For example, aggregation is generally evaluated using size-exclusion chromatography. However, this type of analysis is relatively low throughput and requires quantities of protein beyond what would be desirable for early-stage screening. Fortunately, measurements of the melting temperature (*T_m_*) and aggregation temperature (*T_agg_*), obtained from intrinsic differential scanning fluorimetry (*T_m_*) and near-UV light scattering (*T_agg_*), correlated well with measurements of aggregation during expression, accelerated stability, and particle formation, as well as with various purification properties. Furthermore, high melting and aggregation temperatures were correlated with the best colloidal properties. Using a strategy based largely around early-stage optimization of aggregation behavior, an antibody with a substantially improved developability profile was produced, suggesting that measurements of melting and aggregation temperatures are indeed useful early surrogates for predicting and optimizing some late-stage developability parameters of antibodies [117]. While these findings may have important implications for multispecific antibodies, it is not known if the same correlations apply to such non-traditional formats.

Another powerful way to screen for antibody fragments with favorable developability properties is to use rodents engineered to express human V_H_ repertoires [118]. To realize this goal, transgenic rats containing chimeric heavy chain only antibodies with fully human V_H_ domains have been generated. These transgenic animals are engineered to express a wide range of human sequences that are available from V(D)J recombination and develop large numbers of stable and high-affinity heavy chain antibodies in response to immunization. Such antigen-specific heavy chain antibodies demonstrate favorable stability and aggregation profiles despite the absence of stabilizing V_L_ domains. These uniquely stable V_H_ domains were attributed to specific frameworks and CDRs found in traditional IgGs. The ability to generate highly stable, fully human single-domain antibodies by immunization has tremendous potential for generating antibody fragments with favorable developability profiles for incorporation into multispecific antibodies.

Computation has also been a valuable resource for evaluating the physical processes that lead to aggregation and semi-rational design of aggregation-resistant proteins. The ultimate goal in this area of research is to fully predict antibody aggregation profiles based solely on their primary sequences. One early major step in the computational prediction of antibody properties, including aggregation propensities, was the Developability Index (DI) [119], which is based upon the spatial aggregation propensity (SAP) [96]. This algorithm, which was originally developed using the long-term stability data of a panel of twelve antibodies, correlated antibody hydrophobicity and electrostatic properties, as well as 3D structural information, to aggregation propensity. This enabled the prediction of lead antibodies as well as mutated variants with high stability with respect to aggregation [108,119].

As expected, the generation of more extensive experimental data sets that correlate antibody sequence with aggregation behavior has led to improved computational predictions of antibodies with aggregation-resistant properties. For example, computational methods have been developed for predicting antibody aggregation based upon experimental aggregation data generated from size-exclusion chromatography and an oligomer detection assay [120]. These computational methods, which do not require detailed structural models, were generated using statistical modeling of antibody properties in combination with machine learning. Importantly, these methods were validated by selecting anti-interferon γ (IFN-γ) antibodies that are less aggregation prone and have overall better developability profiles. Although the sequence-based method and the Developability Index method both performed well in terms of predicting antibodies with high aggregation propensity, the former method outperformed the latter one in terms of correctly identifying antibodies with low aggregation propensities, which is important for early-stage antibody screening efforts.

One of the challenges in early-stage screening for aggregation behavior has been the poor correlation between global protein folding stability (as measured by the melting temperature) or fraction of unfolded protein and aggregation at native conditions. This disconnect is attributed to the fact that the dominant aggregation pathway for proteins at native conditions is likely due to local structural perturbations or partial unfolding rather than global protein unfolding. At near-native conditions, local flexible or unfolded protein regions may form intermolecular interactions, leading to protein aggregation without global unfolding. To test this hypothesis, potential aggregation-prone regions that mediate aggregate formation were first identified in the A33 Fab by molecular dynamic simulations and analysis of relevant crystal structure B-factors, which are related to thermal motions of each residue [121]. Rosetta was then used to predict stabilizing and destabilizing mutants, which were tested for their ability to mediate differences in folding cooperativity. As expected, none of the selected single point mutations produced significant changes in the global stability of the Fab, but half of these mutants increased unfolding cooperativity. This cooperativity could be correlated with reductions in conformational flexibility, making it possible to evaluate the role of conformational flexibility in aggregation processes, independent of changes in global stability. The results of this study suggest that aggregation behavior at near-native conditions can indeed be related to short flexible regions of protein structure and that aggregation behavior can be improved by simple mutations that reduce conformational flexibility in these regions.

Although the previous study specifically avoided mutations in the CDRs of the Fab regions in order to preserve the paratope, CDRs are often predicted to be aggregation-prone regions (APRs) [122,123]. These predictions are consistent with findings that show CDRs are often the chief determinants of antibody aggregation behavior [90,91,92,93,119,124,125,126]. Solubis, a computational tool for predicting aggregation propensity of mutants, was used to identify critical APRs in antibodies [94]. This method first uses computational tools to distinguish between structural APRs that are typically buried, and those that are critical for aggregation, by determining their overall contribution to the stability of the antibody as well as their ability to form conformers likely to aggregate without significant unfolding. Once the critical APRs are identified, mutants can be generated that are predicted to both reduce aggregation and maintain the local and global stability of the protein. Interestingly, several of the mutations predicted in this study maintained antigen binding along with their predicted reduction in aggregation behavior. Gratifyingly, the reduction in aggregation propensity also corresponded to an improved expression profile [94].

Despite the significant advances in predicting aggregation propensities of antibodies and antibody fragments, much is unknown about the applicability of these models to multispecific antibodies. For example, most models rely on structural homology modeling or large sets of aggregation data taken from traditional antibody frameworks. In fact, one recent study suggests that the aggregation properties of a multispecific antibody can be more complicated than the sum of its parts [32]. In this study, the levels of aggregation for a Fab attached to a disulfide linked Fv (Fab-ds-Fv) and a Fab attached to a disulfide linked scFv (Fab-ds-scFv) were compared. Note that the first construct involves an Fv fragment stabilized by an additional disulfide bond, while the second construct involves a single-chain (scFv) fragment also stabilized by an additional disulfide bond. These two formats were chosen as simple representations of bispecific formats containing attached Fv or scFv domains. Interestingly, the ranking of the fragments in terms of the % monomer levels of the free ds-Fv and ds-scFv fragments did not correlate with the ranking of the % monomer levels for the corresponding bispecific Fab-ds-Fv and Fab-ds-scFv fragments. This finding implies the distinct nature of “free” versus “tethered” intermolecular association properties and suggests that aggregation screening of multispecifics, at least in some cases, must be conducted in the context of the final antibody format. Fortunately, with advances in multispecific expression and assembly technologies (Section 2.1), it is increasingly possible to produce multispecific constructs en masse. This development, and the high-throughput accumulation of data on multispecific formats, will facilitate the transition from tools that require detailed structural models to those that can make predictions with sequence information alone.

As it is not always possible to engineer multispecific antibodies that do not aggregate, advances in manufacturing have provided an important alternative. For example, flocculation-based pretreatments of mammalian cell cultures have recently been demonstrated as an alternate strategy to reduce aggregates [127]. While the majority of known flocculants adequately remove host cell impurities, one particular flocculant, partially benzylated poly(allylamine) (PAAm-Bn), shows promise in reducing aggregates, in addition to host cell impurities. One study tested the treatment of this flocculant in cell cultures of multiple monospecific and bispecific antibodies, and showed a variable yet PAAm-Bn concentration-dependent removal of aggregates. Multispecific antibodies tend to have higher aggregate levels compared to monospecific antibodies, and therefore require higher concentrations of PAAm-Bn. It is clear that a combination of improved screening, design, analysis and manufacturing procedures will all play important roles in the development of multispecific antibodies with improved aggregation profiles.

### 2.3. Chemical Stability

Although much progress has been made in the efficient assembly of multispecific antibodies with desirable drug-like properties, a somewhat less appreciated barrier to their development as therapeutics is the ability to control and monitor their chemical stability throughout the development process. Chemical stability is of course an essential property for the developability of any therapeutic. In general, the major issues that antibody fragments or multispecific antibodies face (e.g., oxidation, deamidation and isomerization) are also found in traditional IgGs.

**Figure 3:**
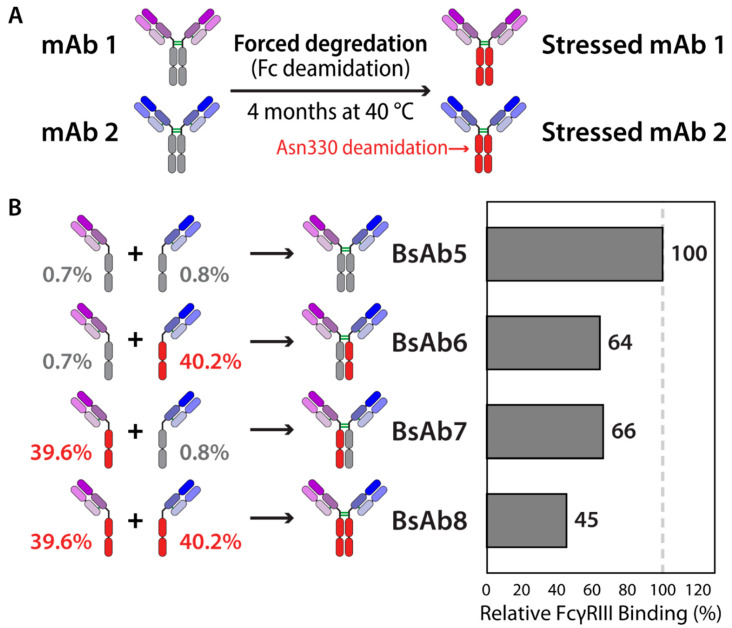
Evaluation of the role of chemical modifications (deamidation) on Fc-mediated receptor binding for bispecific antibodies. Bispecific mAbs were generated with asymmetric levels of deamidation and evaluated for their binding to FcγRIII. (**A**) mAbs 1 and 2 were heat stressed for 4 months to force deamidation in the Fc region (Asn 330). (**B**) Bispecific mAbs with various levels of symmetric (BsAb5, BsAb 8) or asymmetric (BsAb6, BsAb7) deamidation were produced through Fab-arm exchange of pooled native and stressed mAbs. The resulting bispecific antibodies display reduced levels of FcγRIII binding relative to BsAb5 in a manner consistent with total levels of deamidation. The figure is adapted from a previous publication [128].

Although the issues are the same, their study may be complicated by the increased complexity of multispecifics. For example, in order to understand the impact of post-translational modifications on bioactivity, it may be necessary to generate symmetrically or asymmetrically modified antibodies. Even bispecifics based on a traditional IgG scaffold, but comprising of two different variable regions, are likely to contain differences not only in the variable regions but also in the constant regions, which are often mutated to direct heavy or light chain pairing. To address this issue, a strategy based upon the DuoBody platform [63,64] has been developed for generating symmetrically or asymmetrically modified IgG bispecific antibodies (Figure 3). To understand how chemical instabilities impact bispecific antibodies, the DuoBody platform was used to recombine two parental antibodies into a bispecific antibody with high fidelity, such that the parental antibodies could first be modified by chemical degradation (e.g., oxidation or deamidation) [128]. Next, the antibodies were recombined to give four possible variants for analysis (symmetric modified, symmetric unmodified, and both asymmetrically modified bispecific antibodies). Methionine oxidized and asparagine deamidated versions were then used to analyze the effects of such chemical modifications on Protein A, FcRn and FcγRIIIa binding. Interestingly, this study confirmed the proposed stoichiometric binding between antibody Fc and FcγRIIIa receptor, which has implications for predicting the impacts of chemical degradation on multispecific antibody biological activity. Such methods will be critical for evaluating critical quality attributes in IgG-based bispecific antibodies.

Given that the primary analysis technique for most antibody chemical modifications is mass spectrometry, the additional complexity of evaluating chemical modifications in multispecific antibodies is not insurmountable but does complicate the analysis. Fortunately, a combined top-down fragmentation and cation-exchange chromatography method coupled with mass spectrometry has been developed for the site-specific identification of chemical variants that result in charge differences, specifically methionine oxidation. Furthermore, this technology was applied to the analysis of a bispecific antibody [129]. One notable advantage of the top-down methodology is the reduced need for peptide mapping, which is a major drawback of previous strategies. This approach is expected to be useful for future analysis of the impacts of chemical modifications on multispecific antibodies properties.

In addition to the added complexities associated with multispecific analysis, disulfide bond scrambling is another major area where antibody fragments and/or multispecifics present unique challenges. These challenges are especially prominent in multispecifics, particularly those composed of scFvs, because they can also contain additional (engineered) intrachain disulfide bonds that promote domain pairing [130,131,132,133,134,135,136,137]. Mispairing of disulfide bonds can result in complex mixtures and aggregates with reduced activity and/or potential immunogenicity. While many engineering strategies exist to overcome these challenges, a basic understanding of the chemical processes that contribute to mispairing is necessary. Although some of the challenges of intermolecular disulfide scrambling between scFvs were highlighted above, the possible chemical modification of these cysteines through disulfide formation with free cysteine or glutathione can have significant implications for clinical applications and these small modifications can be difficult to detect [138]. Even if the desired intramolecular disulfide bonds are formed during protein expression, reductases found in the harvest cell culture media may promote scrambling by reducing properly formed disulfide bonds [139,140,141,142]. The reduced disulfides can then become mispaired by reoxidation in downstream manufacturing processes [143]. Fortunately, it has recently been demonstrated that high-resolution size exclusion chromatography can, in fact, be used to monitor the levels of undesired species during production [138], which is significant considering the relative similarity of the cysteine or glutathione modified variants to the parental antibodies. This method can be used to accurately monitor these impurities in the production of bispecific IgG-scFvs. However, similar approaches must be extended from traditional IgG-based bispecifics to take full advantage of the diverse array of multispecific formats that have been developed.

## 3. Self-Association and High Concentration Properties

### 3.1. Self-Association and Solubility

The increasing trend toward subcutaneous administration of antibodies requires large quantities of antibodies to be delivered in small volumes [144]. While concentrated antibody formulations provide the benefits of reduced dosing frequency and compatibility with devices used to self-administer antibody-based therapeutics, high concentration formulations also produce crowded environments for antibody molecules. The decreased intermolecular distances and increased molecular collisions in these formulations promotes reversible self-association. Self-association not only contributes to undesirable solubility and viscosity properties but also has the potential to adversely affect shelf-life, pharmacokinetic properties, efficacy, safety and immunogenicity.

The majority of knowledge on antibody self-association is derived from studies of IgGs and efforts to reduce IgG self-association using rational approaches supported by computational insights or predictions. For example, a structural approach was developed to identify sites in antibody variable regions that caused uncontrolled self-association of an anti-nerve growth factor antibody and formation of dimers and higher order oligomers [144]. The researchers mapped the self-association interface to nine amino acids in the V_H_ domain and used computational methods (SAP) [96] to identify solvent-exposed hydrophobic patches. Combining hydrogen-deuterium exchange mass spectrometry data with predictions of aggregation hot spots allowed for the design of a triple mutant with improved solubility [144]. Further analysis of the wild-type and triple mutant antibodies confirmed that the triple mutant primarily existed as a monomeric antibody in solution while the wild-type antibody assembled into oligomeric structures. Furthermore, the triple mutant demonstrated a two-fold improvement in the half-life and decreased nonspecific binding while maintaining high binding affinity.

A separate study on an IgG1 antibody also employed hydrogen-deuterium exchange mass spectrometry in conjunction with structural modelling to identify two regions prone to self-interaction and associated viscous behavior [145]. The investigators identified sites in these regions to mutate to reduce hydrophobicity, including those initially solvent exposed (W50 in V_H_ and Y49 and L54 in V_L_) and solvent shielded (H35 in V_H_). In contrast to the intuitive contributions of solvent-exposed hydrophobic residues to self-association, the effect of buried residues on self-association is more complex. In this case, the buried residue (H35) is positioned within a hydrophobic cavity between V_H_ and V_L_, modifying the local structure and reducing the surface hydrophobicity. While electrostatic interactions typically drive reversible self-association in monoclonal antibodies, this study presents an example in which reversible self-association is predominantly induced by short-range hydrophobic interactions rather than long-range electrostatic interactions. A similar finding was observed for a different monoclonal antibody, containing mutations in the V_H_ and V_L_ regions of the Fab that had a larger contribution to self-association behavior than those mutations in Fc due to their limited surface exposure [108]. These discoveries hold potential for guiding rational design and systematic protein engineering approaches to control self-association behavior in multispecific antibody formats.

One specific result of increased self-association on formulation stability and manufacturing processes is thermodynamically driven liquid-liquid phase separation (LLPS) [146]. The formation of a protein-rich bottom layer and protein-depleted top layer, characteristic of LLPS, typically occurs for highly concentrated solutions at low temperatures (2–8 °C), such as refrigerated formulations [147]. It can also occur during large-scale purification in which intermediates are kept either at room temperature or 2–8 °C for extended periods of time. Low pH elution during Protein A chromatography can also cause LLPS when antibodies elute at high concentration. Although addition of excipients such as sucrose and arginine can prevent LLPS [148], high concentrations of some of these excipients can lead to adverse manufacturing outcomes, such as decreased chromatography performance. Therefore, it would be desirable to minimize LLPS with early-stage property engineering. Recent work has used protein engineering to investigate the mechanistic foundations of antibody site-specific contributions to LLPS behavior [147]. One study found that strong self-association between the light chains of a mAb contributed to electrostatic interactions resulting in LLPS. Homology modeling of the antibody revealed five charged, surface-exposed residues, and AC-SINS and DLS measurements of rationally engineered variants allowed for the identification of key residues responsible for self-association. Disrupting the charge patches or inserting charged residues to increase repulsive interactions substantially decreased LLPS behavior, suggesting that this may be a generalizable engineering strategy.

As the low solubility of antibody fragments often restricts the overall solubility of multispecifics, approaches for studying isolated fragments are advantageous. A majority of preclinical and clinical ocular therapies utilize antibody fragments rather than full-length antibody formats, as their smaller size enables the fragments to cross the cornea in topical applications, which is the preferred route of administration with minimal systemic side effects [149]. Additionally, due to the small acceptable volumes required for both topical and intravitreal administration of antibody-based therapies for ocular targets, such as age-related macular degeneration, the final drug products need to be formulated at high protein concentrations [150]. While antibody fragments are generally less soluble than full-length antibodies, Fab fragments have the potential to be very soluble. One ocular therapeutic against Factor D initially showed limited solubility at low ionic strength, possibly due to weak intermolecular electrostatic interactions that induce assembly of higher-order structures. Although solubility enhancements can be achieved with higher ionic strength formulations that prevent electrostatic association, these formulations can cause adverse reactions, such as detachment and retinal edema [151], which motivates the use of protein engineering strategies to improve solubility. While CDRs are known to play a dominant role in antibody stability and affinity, they may also be large contributors to solubility [90,91,93,99,125,152,153,154]. As such, CDR engineering is a major focus in the development of antibodies with favorable solubility in concentrated formulations. Researchers increased the solubility of anti-Factor D by light chain CDR1 engineering, resulting in a variant that can be formulated at high concentrations (>250 mg/mL) and low viscosities (<17 cP) for intravitreal injection [150]. Importantly, the mutations that improved solubility and stability also retained antigen-binding capacity and favorable PK properties.

**Figure 4:**
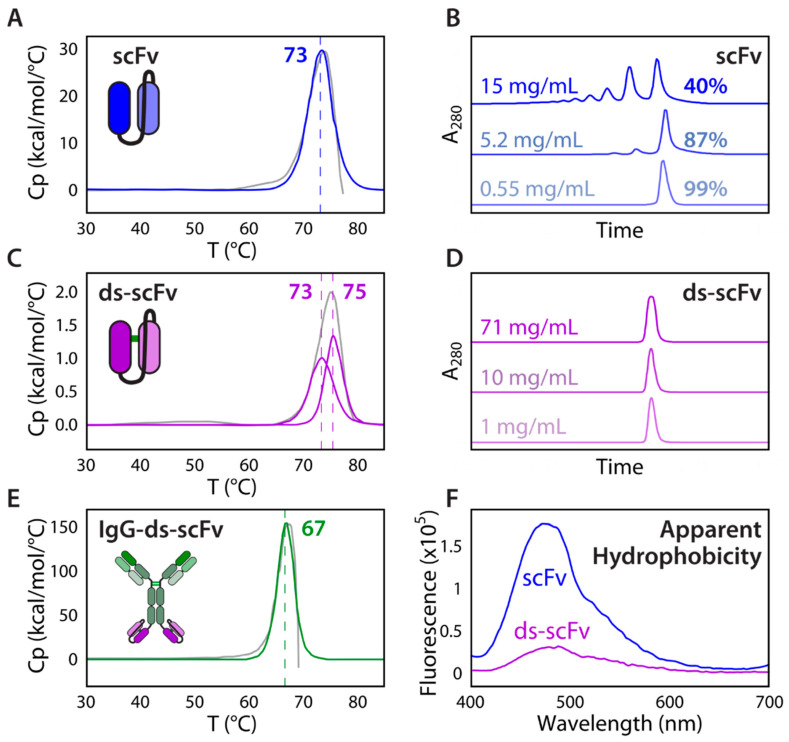
Impact of an additional intramolecular disulfide bond in single-chain antibody fragments, either in isolation or as IgG-scFv bispecific antibodies, on their biophysical properties. Comparison of an anti-IL17 scFv with and without an additional intramolecular (H44-L100) disulfide bond as isolated scFvs or IgG-scFv fusions. (**A**) Differential scanning calorimetry (DSC) thermogram and (**B**) analytical SEC chromatograms for the wild-type scFv. (**C**) DSC thermogram and (**D**) analytical SEC chromatograms for the disulfide-stabilized scFv. (**E**) DSC thermogram of the disulfide-stabilized bispecific antibody. (**F**) ANS fluorescence emission (300 µM ANS) of the wild-type (blue) and disulfide-stabilized (purple) scFvs. In (**A**,**C**,**E**), the raw data are shown in grey, and the fitted data are shown in color (blue, purple or green). The figure is adapted from a previous publication [155].

Rational design of antibody fragments has been successfully used to enhance stability and reduce reversible self-association in a multispecific IgG-scFv (Figure 4). Specifically, an anti-IL17A scFv was grafted onto an anti-BAFF IgG4 heavy chain C-terminus via a glycine-rich flexible linker to create a tetravalent bispecific IgG-scFv [155]. The original IgG-scFv displayed poor biophysical properties, including aggregation and concentration-, pH-, and salt-dependent self-association, which was not observed for the parental antibodies. While the scFv purified at 0.55 mg/mL consisted primarily of monomers, concentration to 15 mg/mL created oligomeric species that dissociated upon dilution, indicating reversible association. Given the high melting temperature of the parental scFv, this reversible self-association suggests that dynamic domain exchange between the V_H_ and V_L_ at high concentrations can cause oligomeric species to form between scFvs in the open configuration. Furthermore, chemical degradation of the scFv attached to the IgG was also observed, including clipping of the anti-IL17A scFv tether, heavy chain oxidation, and light chain deamidation. As these issues were not apparent in the parental anti-IL17A antibody, the poor biophysical properties were linked to the scFv. The scFv was therefore rationally engineered by replacing labile CDR residues with stability-enhancing and affinity-maintaining mutations, balancing the CDR charge distribution to disrupt charged patches, and inserting a H44-L100 disulfide bond at the scFv interface to halt the dynamic domain exchange between the V_H_ and V_L_. This strategy proved highly effective, as the disulfide-stabilized scFv showed no apparent self-association up to 71 mg/mL. Once formulated into the final IgG-scFv, the engineered antibody showed improved physical and chemical stability, as well as decreased aggregation propensity, which could be explained by the lower measured hydrophobicity using ANS fluorescence measurements.

While systematic protein engineering studies are important for understanding the determinants of protein self-association and solubility, there is great need for experimental screening methods that identify highly soluble antibodies early in discovery to reduce the need for protein engineering and formulation development at later stages. To this end, there has been significant effort in developing high-throughput, early-stage experimental methods to identify highly soluble antibodies. To be of maximal utility, these early-stage assays must accurately predict solubility behavior using low protein quantities available at the discovery stage. Assays that measure self-association using small amounts of protein, such as AC-SINS [156,157,158], can be used in the early stages of antibody development to eliminate antibody candidates with high self-association that may display poor solubility. The AC-SINS assay has also been used to characterize unpurified, ultra-dilute mAbs directly in cell culture media, eliminating the need for antibody purification in order to measure self-association [159]. This study tested the self-association propensities of 87 unpurified antibodies and found that extremely dilute solution measurements of self-association (~0.001 mg/mL), as identified from the assay, correlated with high solubility at three to five orders of magnitude higher concentrations (>100 mg/mL). This finding underscores the potential of the AC-SINS assay for measuring antibody self-association in the presence of cell culture contaminants at extremely low antibody concentrations.

More recently, a tour de force study was reported that used a large panel of clinical-stage antibodies and a panel of early-stage assays to evaluate antibody biophysical properties, including self-association, which may be important in the success of antibody therapeutics [116]. Two self-interaction assays, clone self-interaction by biolayer interferometry and AC-SINS, ranked in the top five biophysical assays for identifying drug-like antibodies, second to assays that measured non-specific interactions. A separate, equally impressive study utilized sequence-based data analysis and Pearson and Spearman correlations on early-stage assay measurements of IgG1 and IgG4 antibodies to predict antibody developability [117]. The discovery-stage assays were prioritized into two categories, with a higher priority assigned to assays that determine unacceptable properties. In general, IgG4 antibodies were found to have a greater tendency to self-associate in comparison to IgG1 antibodies. This finding is in agreement with an even more recent study [160], the fact that IgG4 antibodies tend to have lower isoelectric points compared to IgG1 antibodies, and that antibodies with higher isoelectric points generally have improved biophysical properties. Multiple case studies were conducted with the goals of improving aggregation propensity and self-association behavior. For example, an attempted reparation of a heavy chain CDR3 tryptophan oxidation site in a humanized IgG1 kappa antibody (termed wild type) led to the evaluation of ten variants, each with a different mutation at position W104 in heavy chain CDR3 [117]. The W104X variants exhibited comparable measurements to wild type for a majority of biophysical properties except AC-SINS and dynamic light scattering. The W104X variants that showed increased self-association using AC-SINS were also confirmed to display unusually large hydrodynamic radii by light scattering. Only one of these variants (W104F) retained antigen binding, but displayed unacceptably high self-association, preventing its therapeutic development.

Another early-stage screening approach for identifying antibodies that are likely to be well behaved at high concentrations is the addition of crowding reagents such as polyethylene glycol (PEG) or ammonium sulfate to antibody solutions [161,162,163]. These crowding reagents lower the intrinsic maximum protein solubility, which can be evaluated in moderate throughput by quantifying the apparent solubility using a fraction of the amount of protein in comparison to traditional solubility measurements (e.g., concentration by ultrafiltration). A combination of two assays (a vapor diffusion technique and PEG-induced precipitation) carried out in a high-throughput, automated manner has been used to rank order monoclonal antibodies based on their relative solubilities [164]. However, it is unclear whether methods such as PEG-induced precipitation can be used to probe solubility of a wide variety of multispecific formats. For example, in one study, PEG-induced precipitation accurately predicted solubilities of Fc-fusion proteins and mAbs, but was not able to predict solubility of single-domain antibodies [165]. Because methods that use crowding reagents rely on excluded volume effects, it is unclear how useful they will be for evaluating multispecific antibodies.

Given the difficulty associated with measuring reversible self-association, computational methods that can accurately predict this property are highly desirable. However, as reversible self-association can be induced by a sophisticated interplay of hydrophobic and electrostatic interactions, creating prediction algorithms that identify interaction hot-spots is challenging [108]. One key approach for computationally predicting self-association is through quantitative structure-property relationship (QSPR) predictions, a technique that uses scoring functions derived from antibody primary sequence and/or structural descriptors [166,167,168]. However, the accuracy of this method is limited by the composition of the antibodies in the training set, and for novel antibody formats such as those employed in multispecific antibody design, there is no optimal training set currently available.

For these reasons, there is strong interest in improving the current standard of physics-based simulations, which are not limited on training data. Physics-based simulations, however, can be restricted by available computational resources, especially at increasing levels of detail (e.g., fully atomistic). To overcome these constraints, coarse-grain simulations may prove to be a suitable solution. Coarse-grain molecular simulations at the one bead-per-amino-acid degree of coarse-graining have been used to evaluate protein-protein interactions computationally, as this amount of detail was found to best balance trade-offs between accuracy and computational load [169]. The results from these models have been correlated with light scattering data across a range of formulation conditions (pH 5, 6.5, and 8, and ionic strengths varying between 5 to 300 mM) [170]. These models were used to effectively predict the *B*_22_ values of three monoclonal antibodies that exhibit a range of colloidal behaviors, demonstrating the utility of the potential generality of the approach. Further research on solubility predictions using computational methods should address current limitations, such as finite computational resources, and supplement the predictions with accelerated antibody design and experimental assessment of solubility and reversible self-association at the preliminary stages of candidate screening. Models that rely on Lennard-Jones potentials are less effective when evaluating self-association at low concentrations [166]. To overcome this limitation, longer range van der Waals potentials can be employed [171]. A different coarse-grain model that also accounts for electrostatic and hydrophobic interactions was applied to two model mAbs, and while the original model was able to predict the equilibrium self-associated structure, it overpredicted self-diffusivity and underpredicted viscosity. An improvement was observed when the protein clusters in the simulations were rigidified. Future developments in computational modelling should focus on better quantifying the dynamics of closely interacting entities to yield more accurate predictions of self-association.

### 3.2. Viscosity

Viscosity is another property of unique concern for concentrated antibody solutions that affects almost every stage of development, including protein purification, formulation, and delivery. High viscosity is caused by an amalgam of intermolecular interactions, including electrostatic, hydrophobic, dipole-dipole, hydrogen bonding, and van der Waals interactions. The viscosity threshold for subcutaneous injection formulations is less than ~15–30 centipoise [138]. High concentration formulations with increased viscosity above this threshold can make injection either extremely difficult or painful for the patient [149]. Thus, significant efforts are devoted to develop high concentration formulations required for dosing with acceptable viscosity.

To better understand the molecular origins of viscous antibody formulations and particularly for bispecific antibodies, a previous theoretical study used covalently-linked hard spheres with different attractive interactions to model antibodies as Y-shaped molecules [172]. Interestingly, such models show that bispecific antibodies with two different binding arms tend to be less viscous than monospecific IgG, as linkages through a single Fab arm form dimers instead of the higher-order networks formed by bivalent monospecific antibodies with similar Fab arms. However, when three sites are involved, such as in the case of an antibody in which the Fc regions also mediates attractive interactions, the formation of branched clusters generates high viscosities [173,174].

Antibody fragments, in general, do not exhibit viscosity-related issues, because most fragments cannot be concentrated to sufficient levels for abnormally high viscosity and their monovalent formats are less likely to lead to network-like structures often found in highly viscous solutions. In contrast, multispecifics have the potential to demonstrate high viscosity. It has previously been determined that asymmetric distributions of positive and negative charge [175] in antibodies can create dipole-like electrostatic attraction [175,176], increasing reversible self-association and the formation of transient antibody networks associated with viscosity [177].

To further study the impact of asymmetric charge distributions on reversible self-association, researchers evaluated the effects of charge neutralization on mAb solubility and viscosity. Specifically, a mAb known to display electrostatically-driven viscosity was mutated within and near charge patches in the CDRs. The mutations included both charge neutralization (E59Y) and charge reversal (E59K/R) variants [178]. This site (light chain E59) was in one of the largest negatively-charged patches in the variable regions and was located at the outskirts of the antigen-binding site. Interestingly, charge neutralization was able to decrease viscosity while charge reversal increased self-association. This finding is significant as multispecific antibodies may consist of a large number of charged patches, and further investigation on how such patches interact with each other can provide insights into viscosity-reducing strategies for multispecifics. Furthermore, the large variability in multispecific formats may demand unique strategies for controlling viscosity. Nevertheless, some multispecific antibodies do not contain Fc regions, such as bispecific antibody fragments (e.g., BiTES and DARTs), and are expected to display unique viscosity profiles. Another finding from this study was that certain mutations can affect the structure of antibody subunits distant from the mutational site. Such interdomain contacts and allosteric influences may be even more pronounced in multispecific antibodies that consist of a greater number of adjoined fragments. A double mutant (V44K/E59Y) in V_L_ was generated with the E59Y charge neutralization mutation and a second mutation (V44K) that disrupted a solvent-exposed hydrophobic patch [179]. The double mutant exhibited higher apparent solubilities in comparison to the single mutations. Another promising double mutant (V44K/E59S) in V_L_ showed improved properties such as enhanced solubility, decreased viscosity, increased stability, and greater activity. The synergistic effects of the mutations at positions 44 and 59 revealed that hydrophobic and electrostatic interactions work in concert to increase viscosity. For viscous effects that arise from a combination of long-range electrostatic interactions and short-range hydrophobic interactions, understanding the relative contribution of these interactions is important, because a strategy that addresses both issues is often required.

Although electrostatic contributions to viscosity are significant, recent studies have examined the role of other molecular determinants of viscosity, as multispecific antibodies can display complex viscosity behavior that deserves further consideration [174,180,181]. In comparison to monoclonal antibodies, multispecifics can be larger molecules and typically display asymmetrical shape, charge distribution, and hydrophobicity [180]. A study of Dual Variable Domain Immunoglobulin (DVD-Ig) bispecific antibodies was conducted to examine the effect of molecular size, excluded volume, charge patches, and protein-protein interactions on viscosity. The DVD-Ig was considerably more viscous than the mAbs and the effect was pronounced at very low ionic strength (water). In comparison to globular proteins, the Y-shape of monoclonal antibodies leads to higher intrinsic viscosities. The DVD-Ig format consists of additional variable domains that account for a significant increase in the intrinsic viscosity. Excluded volume calculations highlight the significant impact of molecular size on viscosity for high concentration formulations (>100 mg/mL). Multispecific antibodies at pH values near their pIs can also display high viscosity due to attractive electrostatic interactions between oppositely charged patches on their surfaces.

**Figure 5:**
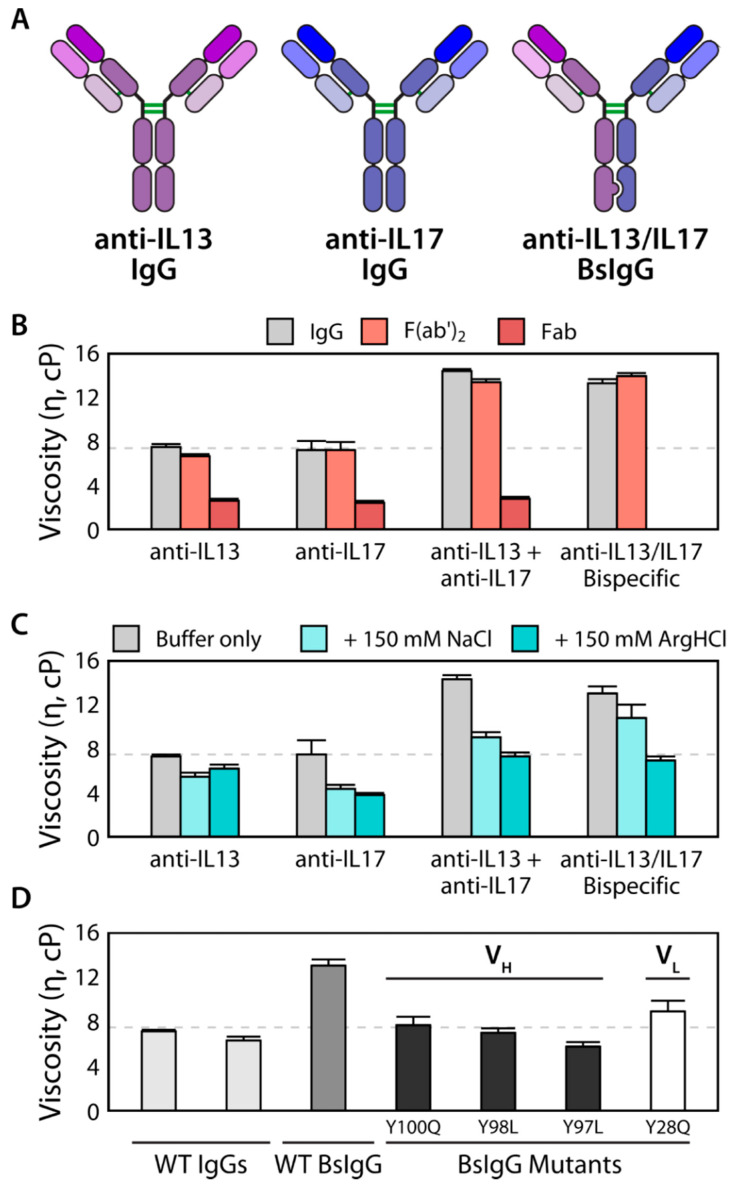
Evaluation of the viscoelastic properties of bispecific antibodies and approaches for reducing their viscosities using formulation and mutational strategies. (**A**) Structures of anti-IL13/IL17 mono- and bi-specific antibodies, the latter of which were generated using knob-in-hole heavy chain pairing methods. (**B**–**D**) Measured viscosities at 150 mg/mL in standard formulation conditions (20 mM His-HCl, pH 6.0) for (**B**) various antibody formats, (**C**) IgGs at different formulation conditions, and (**D**) IgGs with various point mutations in the anti-IL13 variable domains. The viscosity of one of the parental IgGs is shown with a grey dotted line. The figure is adapted from a previous publication [174].

In the process of studying the viscosities of multispecific antibodies, the contributions of additional intermolecular forces, apart from electrostatics and hydrophobic interactions, have been elucidated. For instance, the anti-IL13/IL17 bispecific IgG4 antibody displayed a significant increase in viscosity compared to the parent monospecific antibodies (Figure 5) [174]. Interestingly, a 1:1 mixture of the two parental antibodies (anti-IL13 IgG4 and anti-IL17 IgG1) also exhibited increased viscosity. A further investigation showed that bivalency is required for network formation and high viscosity solutions. To determine the contribution of different intermolecular interactions to reversible self-association, viscosity was measured under different solution conditions. Arginine-HCl and guanidine-HCl excipients were more effective than NaCl at decreasing viscosity by reducing intermolecular interactions, revealing the significant contributions of cation-π and π-π interactions relative to electrostatic interactions. This is in agreement with previous computational studies that suggest cation-π interactions are stronger than analogous salt bridges in aqueous solutions [182]. Molecular dynamics simulations confirmed that arginine significantly interacts with solvent-exposed aromatic residues and mutation of such CDR residues led to the discovery of two anti-IL13 variants with reduced viscosities that were similar to those of the parental antibodies [174]. This case study corroborates the current theory that interactions between different Fab regions can form networks of bivalent molecules, leading to increased viscosity. Moreover, this study highlights the potential importance of cation-π and π-π interactions in mediating viscous solution behavior.

The use of antibody mixtures to predict the viscoelastic behavior of bispecific antibodies has also been reported in another study [181]. The investigators compared viscosities of equimolar mixtures of two sets of parental mAb pairs and their bispecific counterparts, and compared these measurements to predictions of viscosities of mixtures using the Arrhenius mixture model. This study consisted of a slightly viscous antibody (mAb A), a highly viscous antibody (mAb C), and its bispecific counterpart (BsAb A/C). The viscosity of the bispecific variant was similar to the 1:1 mixture of each mAb, as the two parental mAbs participate in similar protein-protein cross interactions. A similar experiment was carried out with a separate set of antibodies that displayed similar viscosity profiles and significantly different isoelectric points (pI 6.3 for mAb A and 9.3 for mAb B). Because mAb A and mAb B have dissimilar intermolecular interactions, the mixture and bispecific antibody displayed much higher viscosities than the individual mAbs and deviated significantly from the Arrhenius mixing prediction. Interestingly, Rayleigh scattering experiments indicated a large contribution of short-ranged, non-electrostatic attractive interactions to the observed viscoelastic behavior in addition to the expected contribution of attractive electrostatic interactions.

As demonstrated in diverse previous studies, predictions of diverse antibody biophysical properties rely on trends that are gathered from experimental data [116,117]. A recent study on engineering antibodies with reduced viscosities extensively sampled Fv and CDR charge mutations and measured the viscosity of 40 unique variants [183]. The viscosity of the parental antibody was 40 cP (measured) at ~100 mg/mL and 250–300 cP (extrapolated) at 150 mg/mL. In order to be suitable for subcutaneous injections, the viscosity needed to be lowered to below 20 cP for a 150 mg/mL antibody formulation. Two rounds of structure-based design allowed for the identification of a variant that met these criteria while maintaining binding affinity. The most promising mutations were located in a negative charge patch in the middle of light chain CDR2. Furthermore, because charge asymmetry is influenced by individual residues, computational models that only use domain-level charges were not able to discriminate the differences in viscosity of the antibody variants in this study. It is also important to note that the rational design process benefited from the accessibility of an X-ray crystal structure, as this structural information greatly reduced the total mutational space that needed to be scanned.

Given the challenges in experimental viscosity analysis due to unrealistic requirements for large (mg) amounts of antibody, computational methods for predicting antibody variants with low viscosity are particularly valuable. One proposed computational method is referred to as the spatial charge map (SCM) method, which uses antibody sequences and corresponding homology models to detect highly viscous antibodies at the initial stages of the development process [184]. Analysis is only performed on the Fv region, which contains the majority of the variability between antibodies. The SCM tool uses hydrophobicity (hydrophobicity index), net charge, and charge asymmetry properties to generate an SCM score. This method has been validated using multiple panels of IgG1 antibodies, and the model is able to accurately identify highly viscous antibodies. It should be noted that solution conditions, such as buffer components and temperature, are not accounted for in the analysis. Therefore, the ability of the SCM score to predict high viscosities can vary and is expected to be more successful when comparing antibodies of the same isotype at similar formulation and temperature conditions. Future work is needed to not only account for different formulation conditions but also for applications to multispecific antibodies.

Similar to solubility predictions, coarse-grain models can also be used to predict viscosity [166]. A recently developed, novel coarse-grained simulation method analyzes molecular dynamics simulations of highly concentrated full-length antibodies at the microsecond time-scale. This new method differs from previous coarse-grain simulation methods in that the model accounts for the high-order electrostatic multipole moments, charge asymmetry, and hydrophobic character of full-length antibodies, all of which are factors that contribute to self-association. High-order electrostatic interactions, which are generally negligible in dilute conditions, are significant at high concentration, and the notably different electrostatic potentials between mAbs can lead to differences in viscosity. However, the importance of accounting for hydrophobicity in the viscosity predictions should not be underestimated, as evidenced by antibodies that show minimal differences in their electrostatic fields and multipole moments but large differences in their hydrophobicities. Accurate values for both the hydrophobicity and electrostatic components are also necessary when accounting for the impacts of changes in ionic strength on the viscosity of mAbs in different formulations, as the viscosity of concentrated antibody solutions is generally mitigated by the addition of salt and excipients [185,186,187,188].

As molecular crowding also influences the diffusion of antibodies in solution, diffusivity measurements can be used to assess intermolecular interaction strength that is often inversely correlated with viscosity. This novel coarse-grain model was applied to a set of fifteen antibodies, and the results highlight the influence of intermolecular interactions on the diffusion coefficients of antibodies in crowded environments [166]. Interestingly, an inaccurate prediction of a highly viscous antibody led to further investigation on the causes behind the inconsistency. The calculated modest hydrophobicity and comparatively large net charge anticipated repulsive intermolecular interactions and therefore low viscosity. The discrepancy is speculated to arise from errors in homology modeling, particularly those related to heavy chain CDR3 loop, as the crowded environment in viscous, high-concentration formulations may destabilize the antibody structure and alter the antibody conformation.

Coarse-grain simulations have also been used to further understand the molecular basis for antibody network formation and discern whether the number or frequency of domain-domain interactions are linked to greater antibody self-association [177]. Unlike previous simulations, the use of an asymmetric mAb in which one Fab arm is located much closer to the Fc fragment compared to the other Fab arm showed that the asymmetry significantly impacts the prevalence of intermolecular interactions. Inspiration for the modeling of an asymmetric mAb was derived from the preferred conformation of an HIV-1 neutralizing IgG1 antibody in solution, in which the non-flexible asymmetric shape is a property inherent to the antibody [189]. As many multispecific antibodies are asymmetric by design, this model can potentially be extended to such entities as well. Researchers have used this model to quantify features of mAb network formation for high and low viscous mAbs at different concentrations [177]. In general, viscous antibodies with more negatively charged Fv regions tend to have more nodes, defined as mAbs that are in contact with three or more mAbs (closer than the 35 Å cut-off), and the number of nodes increases with concentration. These models indicate that a larger network density (correlated with high viscosity) allows for a greater stress-bearing capacity and increased structure that withstands deformation under shear stress. Complementary charges between mAb domains increase viscosity by slowing molecular mobility, which is reflected in smaller diffusion coefficients for the highly viscous antibodies. In fact, domain-domain interactions play a greater role in comparison to contact frequency in highly viscous mAbs at the same concentration. Although the coarse-grain model can be improved by incorporating the effect of solvent on network formation (a feature that was omitted due to computational resource constraints), the study corroborates the use of coarse-grained simulations to predict viscosity. This newfound understanding of the molecular determinants of viscosity may hold promise for the prediction and design of multispecific antibodies with favorable viscoelastic properties.

## 4. Polyspecificty and In Vivo Properties

### 4.1. Polyspecificity and Pharmacokinetics

Since pharmacokinetic (PK) properties such as systemic clearance, volume of distribution, and half-life can influence efficacy and toxicity, accurate measurement of these properties is critical. Due to the low-throughput nature of many PK profiling strategies, PK properties are typically evaluated in the late stages of antibody development, presenting a considerable bottleneck to the development process. Although the full PK profile of an antibody is the result of a complex set of processes including non-specific binding, immunogenicity, and FcRn recycling, polyspecificity is a key contributor to poor antibody PK behavior because non-specific binding can lead to a number of unfavorable PK properties, including short half-lives and increased toxicity. Most of the available antibody polyspecificity data is for IgGs. Interestingly, a recent study of IgGs revealed that approved antibody therapeutics are generally more specific and display lower levels of non-specific binding than those in clinical trials, suggesting that high specificity may be a critical attribute of drug-like antibodies [116].

Current in vitro methods for measuring antibody polyspecificity, as reviewed elsewhere [190], involve evaluation of antibody interactions with polyclonal antibodies (cross-interaction chromatography) [191], baculovirus particles (BVP assay) [192], a combination of protein and non-protein antigens (DNA, lipids, proteins, and polysaccharides; ELISA) [193] or soluble membrane and cytosolic proteins (polyspecificity reagent, PSR assay) [116,194]. These types of non-specific reagents, such as PSR, are even being employed during the antibody discovery stage using in vitro high-throughput methods, such as yeast surface display, to select antibody candidates with low polyspecificity [194,195,196,197,198]. Removing nonspecific binders in the library-screening stage of antibody discovery prevents the arduous process of rectifying abnormal PK properties due to off-target binding for late-stage antibody candidates. The PSR reagent can be prepared using soluble membrane and cytosolic proteins obtained from either CHO or HEK cells [194]. This reagent is biotinylated and subsequently incubated with antibody-presenting yeast. Antibody binding to PSR can be probed via flow cytometry in a high-throughput manner (10^8^ cells/h). Importantly, the PSR assay has been shown to correlate with antibody clearance rates in mice [195]. However, one drawback of using PSR to probe polyspecificity is that, due to the ill-defined nature of the reagent, lot-to-lot variability of the complex cell extract can lead to different assay results. The need for well-characterized, defined reagents has led to the use of Hsp90 and insulin as single-component specificity reagents that also correlate to antibody clearance rates in mice [196,199]. Previous specificity assays, employing microarray technologies or microtiter plate assays, afford much lower throughput (tens to hundreds) relative to FACS-based approaches that can process millions of variants [200].

Increased antibody non-specific binding in general, and in particular to FcRn, is generally linked to increased risk of fast antibody clearance [192,199,201,202,203,204,205]. Therefore, it is necessary to both obtain accurate PK measurements in a high-throughput manner at earlier stages of development and establish in vitro/in vivo correlations (IVIVC). In a recent study, researchers formulated a combinatorial triage approach to differentiate antibodies with favorable and unfavorable PK profiles [199]. This strategy involved a set of five in vitro assays that could be grouped into three categories—nonspecific binding assays (DNA- and insulin binding ELISAs), a self-association assay (affinity-capture self-interaction nanoparticle spectroscopy, AC-SINS), and two assays to measure interactions with matrix-immobilized human FcRn (surface plasmon resonance and column chromatography). Results showed a group of antibodies with low clearance and assay scores, enabling the demarcation of a clearance threshold at ≥7.7 mL/day/kg. Human clearance values were estimated by allometrically projecting clearance values measured in Tg32 mice. A threshold value for each in vitro assay was defined by applying this clearance threshold to each mAb set, and antibodies that scored below the threshold in all three categories were deemed to have favorable PK and those that scored above the threshold in all three categories were rejected due to unfavorable PK. The PK properties of antibodies with above-threshold scores in one or two assay categories were further investigated in mice. Notably, the correlations of these in vitro assays to allometrically projected human clearance based on mice clearance values were statistically significant. In another study, a novel assay was developed for PK analysis of a fully human anti-EGFRvIII/anti-CD3 bispecific T-cell engager (BiTE) using microflow liquid chromatography coupled to high resolution parallel reaction monitoring mass spectrometry [206]. Using this method it is possible to quantify the bispecific antibody levels in mice plasma and whole blood.

However, even early stage high-throughput screens for non-specific binding, which is only one property linked to antibody PK, can be time and resource intensive. The significant costs incurred from realizing unfavorable PK properties in late-stage antibody candidates incentivizes the use of predictive methods at even earlier stages of development, such as at the stage of in vitro antibody discovery. Advances in this area by several research teams have paved the way for generalized predictions of problematic amino acid motifs and nonspecific antibodies in general. While it has long been known that over enrichment in positive charge and hydrophobicity can lead to nonspecificity [144,197,207,208,209,210], one study used molecular modelling to determine residue substitutions in the CDRs that disrupt positively charged patches [211]. They were able to reduce the net positive charge of the Fv region without affecting the overall antibody pI, although the effect was more significant for IgG4s in comparison to IgG1s. In a separate study, panning nonimmune yeast display libraries elucidated the dominant role of a constrained β-sheet structure in heavy chain CDR2 of V_H_6 antibodies in driving nonspecificity [198]. Furthermore, homology modeling showed that an Arg50 residue at the base of this CDR stabilizes the β-sheet and rigidifies the structure of this loop, allowing it to protrude farther than other heavy chain CDR2 loops. The exposure of other residues in the same loop could also account for the increased polyspecificity. This finding can be used to exclude V_H_6-1 variants during future library generation. Other studies employing synthetic yeast libraries have identified motifs containing Trp, Val, and Arg in the center four positions of heavy chain CDR3 of nonspecific antibodies [197]. In order to further develop our understanding of nonspecificity motifs, approaches that combine next generation sequencing, homology modeling, and machine learning will be required in the future.

Computational models provide an alternate method to investigate sequence determinants of specificity. Analysis of published non-specific interaction data for clinical-stage antibodies revealed a strong positive correlation between non-specific binding and increasing numbers of arginine and lysine residues in antibody CDRs [212]. Conversely, the investigators found a strong negative correlation between non-specific binding and increasing numbers of aspartic acid and glutamic acid residues. These results further showed that arginine, in comparison to lysine, plays a greater role in mediating nonspecific interactions, and aspartic acid, in comparison to glutamic acid, plays a larger role in mediating highly specific interactions. In an effort to identify highly specific antibody candidates based primarily on antibody sequences, chemical rules based on physicochemical properties of different regions in antibody variable fragments have also been recently established [213]. Combinations of these chemical rules could be used to effectively identify antibodies with improved specificity during early-stage discovery and protein engineering.

Monoclonal antibodies typically exhibit slow systemic clearance and low volume of distribution (Vd) as their size and polarity confines their distribution to the vascular and interstitial spaces [199,214]. These two factors, combined with FcRn-mediated recycling, result in the prolonged half-life of mAbs. PK properties of mAb-based therapeutics can vary based on their nature (humanized vs. human), type (IgG1, 2, 3, or 4) and mode of binding (monospecific vs. multispecific) [215]. Systematically administered mAbs generally show biphasic PK profiles, characterized by a fast distribution phase followed by a slower elimination phase [214]. The general consensus for the threshold value that constitutes fast antibody clearance varies between >7.7 mL/day/kg to >20 mL/day/kg [192,195,199]. These clearance threshold values are determined from a variety of factors, including analysis of receiver operating characteristic (ROC) curves and allometric scaling of clearance thresholds values previously reported in cynomolgus monkeys. Clearance pathways for antibody therapeutics that are larger than ~55 kDa (the glomerular filtration threshold) are typically through pinocytosis and proteolysis (nonspecific), target-mediated specific clearance, and ADA-mediated clearance [214]. Multispecific antibodies have a higher risk for displaying anomalous PK properties, such as shorter half-lives and increased clearance rates compared to monoclonal antibodies [216,217,218,219].

**Figure 6:**
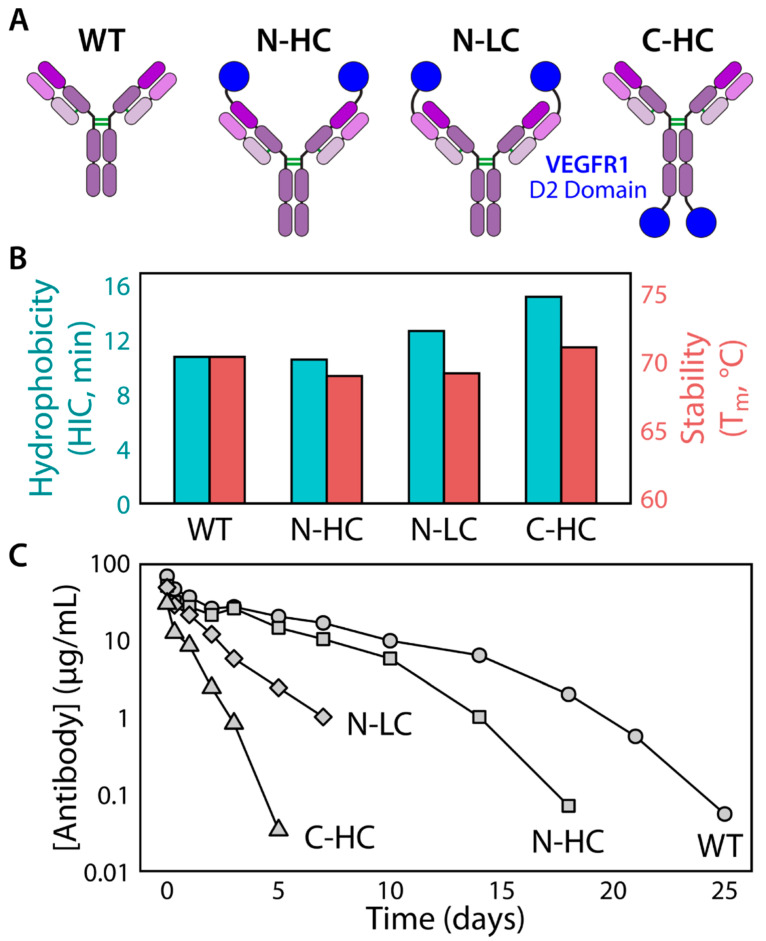
Effect of molecular architecture of IgG fusion proteins on their hydrophobicities, stabilities, and pharmacokinetic properties. (**A**) Representations of various IgG-extracellular domains (ECDs) with the D2 domain of VEGFR1 fused to different regions of the wild-type IgG. (**B**) Hydrophobicity measured by hydrophobic interaction chromatography (HIC; blue) and stability (first thermal unfolding transition, red) of the wild-type (WT) IgG and IgG-ECDs. (**C**) Pharmacokinetic profiles of the WT IgG and IgG-ECDs in cynomolgus monkeys (single 2 mg/kg iv dose) reveal increased clearance rates for more hydrophobic formats. The figure is adapted from a previous publication [219].

One notable study evaluated the PK properties of a bispecific fusion protein in which a biologically active protein domain (termed extracellular domain or ECD) was attached to an IgG4 antibody via a flexible linker (Figure 6). This study evaluated the effect of the location of the fusion protein relative to the IgG (e.g., N-terminus of heavy chain) on its biophysical, biochemical, and PK properties [219]. The protein domain was attached to the N- and C- termini of the heavy and light chains of an IgG4 monoclonal antibody. The C-terminal light chain fusion construct was excluded from further studies due to poor expression. Notably, attachment of the protein domain to the N-terminus of the heavy chain (N-HC) lead to a variant with similar hydrophobicity as the wild type, while the other fusions [C-terminus of heavy chain (C-HC) and N-terminus of light chain (N-LC)] displayed higher hydrophobicity. Moreover, PK analysis of these constructs using cynomolgus monkeys and CD-1 mice revealed the least hydrophobic fusion protein (N-HC) displayed PK properties that were closest to the wild type, while the more hydrophobic variants displayed significantly worse PK properties. Given that the bispecific constructs were composed of the same IgG and fusion protein, the differences in hydrophobicity and PK properties demonstrate the importance of molecular architecture on the pharmacokinetic properties of bispecific antibodies.

Multispecific formats can also be constructed to promote favorable PK properties, including longer half-lives [220]. For example, albumin-binding multispecific antibodies that extend systemic half-life have been reported [221,222,223,224,225]. Moreover, application of this concept to ocular therapies in an effort to reduce dosing frequency is particularly advantageous, as each intravitreal injection can expose the patient to a variety of complications, including vitreous hemorrhage, endophthalmitis, and uveitis [226]. In one study, researchers attached a 97-amino acid, hyaluronan-binding peptide to therapeutic antibodies and proteins [220]. Hyaluronan was chosen as the target over other vitreous components, such as collagen type II and albumin, based on three main criteria: (1) sufficient concentration and exposure of binding sites of a vitreous component to effectively bind a clinically relevant dosage of drug; (2) low concentration of vitreous target in non-ocular tissues and blood to minimize systemic retention; and (3) low turnover in the eye to reduce target-mediated drug clearance. Fusion proteins consisting of the hyaluronan-binding peptide and anti-VEGF therapeutics [ranibizumab, bevacizumab, aflibercept, NVS0 (Fab), and brolucizumab (scFv)] were injected into the vitreous of rabbits and cynomolgus monkeys. Fusion of the hyaluronan-binding peptide did not hamper the ability of anti-VEGF therapeutics to neutralize VEGF in vivo. In fact, a key finding was that reduced doses of these fusion proteins (as much as 50-fold lower) can demonstrate either commensurate or superior activity in comparison to the parental anti-VEGF antibody while exhibiting the desired half-life extension that enables reduced dosing frequency. The potential generality of using this type of approach in diverse multispecific antibody formats holds great therapeutic promise.

### 4.2. Immunogenicity

Another key challenge in developing antibody therapeutics is their potential immunogenicity [227,228]. This immunogenicity may be mediated by T cells, which recognize short peptide sequences displayed by MHC class II proteins on antigen-presenting cells, or B cells, which produce anti-drug antibodies capable of recognizing conformational epitopes on therapeutic proteins [229,230,231]. A variety of methods exist for determining the potential immunogenicity of protein therapeutics, although the measurement of anti-drug antibodies (ADAs) is the most common [228,231,232,233,234].

ADAs can impact the function of a drug in multiple ways [235]. One of the chief concerns with ADAs is the potential for neutralization of the activity of therapeutic antibodies by interfering with antigen binding. However, even ADAs that do not neutralize antigen binding may otherwise impact the pharmacodynamic or pharmacokinetic behavior of the drug. In addition, ADAs can elicit a potentially dangerous hypersensitive immune response. Therefore, immunogenicity is a primary concern for the development of safe and effective biologics. To this end, a number of strategies have been developed for reducing unwanted immunogenic activity of biologics [236,237], and the FDA has recently released guidance for developing and validating assays to assess immunogenicity of therapeutic proteins [234].

Multispecific antibodies may be particularly susceptible to immunogenic liabilities [238,239]. This is because divergence from the natural human antibody repertoire generally increases the potential for immunogenicity and multispecific antibodies are likely to include multiple unnatural features. For example, multispecific antibodies may contain sequences of non-human origin through the incorporation of antibody fragments such as scFvs or nanobodies. Although such fragments are generally considered to be of low risk for immunogenicity, both could theoretically represent risk factors for immunogenicity due to their linkers and, in the case of nanobodies, non-human origin [240,241]. Even in cases where scaffolds contain fully human sequences, changes in format, such as single-domain antibodies or scFvs derived from mAbs, can lead to exposure of epitopes that are normally buried in the parental mAb and which result in an immunogenic response. Additionally, upon antigen binding, a novel surface, known as a neoantigen, is created at the new binding interface that may be recognized by ADAs. In some cases, immunogenic response to one portion of a multi-domain drug can lead to a phenomenon known as epitope spreading where an immune response to a highly immunogenic subunit results in further responses to otherwise non-immunoreactive portions of a therapeutic. Given the multi-domain nature of many multispecific antibodies, this phenomenon may be of particular concern. The challenges associated with assembly and purification of multispecific antibodies may also result in an increased risk of immunogenicity. Moreover, critical quality attributes like aggregation, particle size, and polyspecificity are also thought to increase the likelihood of immunogenicity [88,89,239,242,243,244]. Given the unique immunogenic liabilities of multispecific antibodies and their increasing numbers in clinical trials, new tools for the analysis and reduction of immunogenic liabilities will be of increasing importance.

Typically, immunogenicity is measured late in the development process using immunosorbent assays to detect ADAs. Although there are some notable limits to these assays associated with interference or quantitation, which have been extensively reviewed elsewhere [233], they are particularly useful for qualitatively determining if ADAs are created in response to a therapeutic. It is also possible to determine the domain specificity of ADAs by evaluating antibody binding to individual domains of a therapeutic, a strategy that has been successfully adapted for use in determining ADA specificity in bispecific antibodies [245]. Interestingly, similar assays have been used to show that pre-existing ADAs for an IgG-scFv corresponded to the same domain as emergent ADAs for the same therapeutic, suggesting that the presence of pre-existing ADAs can be used to predict and even identify regions of immunogenic liability in multispecific antibodies [246]. Despite the utility of these assays, they require human testing, and are limited in their ability to quantify ADA interactions or precisely determine ADA epitopes [247].

To address these issues, a variety of approaches have been developed [228], including MHC-associated peptide proteomics (MAPPs) [248,249]. In this in vitro technique, human professional antigen-presenting cells are incubated with the biologic of interest, the biologic is then internalized, processed enzymatically, and presented at the surface by human leukocyte antigen (HLA) class II molecules. The antigen-presenting cells are then lysed and the HLA class II receptors are isolated by anti-HLA antibody coated beads. The antigenic peptides from the biologic of interest are then released from HLA by reducing the pH. The resulting antigen peptide sequences can be determined by LC-MS. Although these methods are highly validated for identifying immunogenic sequences, the assays require analysis of the blood from at least 20 donors to cover the processing and display variability that is often observed for antigen-presenting cells obtained from different individuals. Therefore, a full analysis can take up to eight weeks for approximately ten therapeutic candidates [249]. Although this method is valuable for determining antigenic liability of biologics without dosing human subjects, MAPPs assays are not practical for early-stage development decisions, as a consequence of the time-intensive and low-throughput nature of this approach.

Fortunately, for some biologics, such as camelid nanobody (V_H_H) domains, it is possible to predict a higher-than-normal immunogenicity risk to humans without sophisticated analytical or computational methods. In such cases, a variety of approaches have been used to engineer out immunogenic properties in the late stages of antibody development, but one recent study described a strategy for engineering out immunogenic liabilities at the initial discovery stage. Specifically, the investigators sought to develop neutralizing single-domain human antibodies for the SARS-CoV-2 coronavirus [250]. Such single-domain antibodies have a variety of advantages over traditional antibodies such as ease of expression in bacterial cells and the ability to be administered to the lung by inhalation. However, single-domain human antibodies have not found wide application due to their poor biophysical properties in the absence of light chains [41]. Instead, nanobodies produced in camelid species make up the vast majority of reported single-domain antibodies. Because these species produce heavy chain only antibodies, their variable regions show superb stability and solubility when expressed in the absence of the conserved domains. Unfortunately, their camelid origin also increases their risk for inducing immunogenic responses in humans. To address this issue, investigators have screened human frameworks for stability, expression level, and Protein A binding [250]. Next, they created a library of human single-domain (heavy chain) antibodies by grafting human CDRs onto the preferred framework. This framework was then used to produce highly stable human single-domain antibodies for a variety of antigens, including the SARS-CoV-2 spike protein. Given the number of nanobody-based multispecific formats, this work is likely to have implications for multispecific antibody generation in the future.

Of course, not all immunogenic liabilities are as easily predictable as those for the camelid nanobodies. In these cases, new strategies to either predict or efficiently screen for protein sequences and structures with immunogenic liabilities would be highly desirable. In addition, once the liabilities can be efficiently identified, robust strategies for improving these liabilities early in screening or development are critical. For mAbs, there is a long history of successful humanization strategies [251,252,253]. For unconventional multispecific scaffolds, computational approaches may play a significant role in addressing both of these hurdles. For example, because immunogenicity arises from non-self-recognition, one major approach to predicting immunogenicity is to evaluate humanness of antibodies. Facilitated by the recent increase in next-generation sequencing data for B-cell repertoires, multiple approaches have recently been reported that demonstrate high fidelity predictions of antibody humanness [254,255]. Although it is now possible to clearly distinguish between therapeutic antibodies in terms of their degree of humanness, it is not clear how these methods will translate to multispecific antibodies.

Another way to evaluate the immunogenic liabilities of protein therapeutics is to predict the peptides that are likely to be displayed on MHC proteins and lead to immune responses. To this end, much effort has been directed toward developing algorithms to predict peptide presentation of MHC class I and MHC class II proteins [256]. These efforts began with peptide binding affinity measurements for MHC proteins, but have been improved by the inclusion of large mass spectrometry datasets from MAPPs-like experiments [257,258,259]. However, deconvolution of this data is difficult for many reasons, but particularly because the number of MHC alleles makes it is difficult to trace any given peptide to a specific MHC protein. Without that critical piece of information, it is difficult to develop a model for MHC peptide binding. To solve this problem, a variety of innovative approaches have been explored, the most recent of which is the development of artificial neural networks that can simultaneously analyze the mass spectrometry data while developing individual MHC binding models [257,260]. The authors report that this strategy results in an immunogenicity predictor that met or exceeded the predictive capacity of previous methods in their test system. Interestingly, this method also identified two regions with predicted immunogenicity in their test set that were not identified by MAPPs analysis, both of which ultimately showed immunogenicity in CD4 T-cell activation assays, suggesting that this method may be able to enhance or improve upon MAPPs analyses. In addition, because the MAPPs data can be generated for biologics in any format, these methods may hold the greatest potential for predicting multispecific antibody immunogenicity in the near term.

## 5. Conclusions and Future Directions

Multispecific antibodies present a number of advantages in terms of their biological activities and, therefore, it is likely that interest in using them in diverse therapeutic applications will increase in the coming years. To address the increased physical and chemical liabilities associated with such non-natural antibody formats, there are a number of challenges discussed in this review that must be met to advance their widespread application. In particular, it will be important to continue to improve expression and purification methods for simplifying the production of multispecific antibodies with high yields and minimal amounts of mispaired or incompletely assembled antibodies. This issue has greatly limited the development of multispecific antibodies and progress in the field in generating them in a rational and systematic manner. Given the vast number of possible multispecific antibody formats, it will also be critical to improve computational methods for predicting antibody variants with favorable biophysical properties to reduce the design and protein engineering space that needs to be explored to identify drug-like variants. This will require large experimental datasets to be generated for multispecific antibody biophysical properties, at least for a subset of the most promising formats, to train and optimize such predictive methods. The importance of such datasets, which are challenging and costly to generate, should not be underappreciated, as the field is unlikely to significantly advance without them due to the lack of systematic design and engineering strategies. It will also be critical to better define the immunogenicity risks of different multispecific antibody formats, which are inherently more likely to be recognized as foreign by the immune system, and improve the prediction of multispecific variants with low immunogenicity. Current methods are limited, even for analyzing immunogenicity risks for conventional IgGs, and significant advances are needed to reduce safety and efficacy risks associated with multispecific antibodies. Despite these challenges, it is likely that the rapid progress toward improving the prediction and identification of drug-like monospecific IgGs in the last decade will continue to inspire and guide related efforts to improve computational and high-throughput experimental developability analysis of multispecific antibodies with unprecedented therapeutic activities.
